# Evaluation of a Pathway team for homeless mental health in-patients

**DOI:** 10.1192/bjb.2022.61

**Published:** 2023-10

**Authors:** Alex D. Tulloch, Zana Khan, Nigel Hewett, Sophie Koehne, Ranga Rao

**Affiliations:** 1 South London and Maudsley NHS Foundation Trust, London, UK; 2 King's College London, UK; 3 Pathway Charity, London, UK

**Keywords:** Homelessness, community mental health teams, in-patient treatment, outcome studies, primary care

## Abstract

**Aims and method:**

The Pathway model is an enhanced care coordination model for homeless people in hospital. We aimed to evaluate the first attempt to apply it on psychiatric wards, which started in 2015 in South London. We developed a logic model which expressed how the Pathway approach might work. Two predictions from this model were tested, using propensity scores and regression to estimate the effect of the intervention among people who were eligible for it.

**Results:**

The Pathway team theorised that their interventions would reduce length of stay, improve housing outcomes and optimise the use of primary care – and, more tentatively, reduce readmission and emergency presentations. We were able to estimate effects on length of stay (−20.3 days; 95% CI −32.5 to −8.1; *P* = 0.0012) and readmission (a non-significant reduction).

**Clinical implications:**

The marked reduction in length of stay, explicable in terms of the logic model, constitutes preliminary support for the Pathway model in mental health services.

People experiencing homelessness – even if ‘hidden’ – have worse health and higher mortality than people in stable accommodation.^1^ Their use of health services also differs: they make less use of scheduled and preventive care,^2^ but attend accident and emergency departments five times as often, are admitted three times as often and stay three times longer when in hospital.^3^ Since 2009, the UK homeless healthcare charity Pathway and the Faculty for Homeless and Inclusion Health have promoted the development of teams that work to improve the health and housing outcomes of homeless people in acute hospitals.^4^ One such team developed to cover the acute hospitals that form part of the King's Health Partners (KHP) Academic Health Sciences Centre and serve the London boroughs of Lambeth and Southwark. In 2014, funding was made available to extend this service to the adult mental health wards for Lambeth and Southwark, which are situated at the Maudsley Hospital and at Lambeth Hospital. These hospitals are operated by South London and Maudsley NHS Foundation Trust (SLaM), also part of KHP. As this was the first attempt to apply the Pathway approach to mental health services, some funding was also provided for evaluation.

## Method

### Logic modelling

The KHP Pathway Homeless Team was treated as a programme: that is, a set of resources and activities deployed with particular aims. Rather than use a ‘black-box’ approach to programme evaluation, we used logic modelling.^5^ This approach turns the situated knowledge of those involved in a programme into a basic theory of programme operation, which is represented as a set of inputs, activities, outputs and outcomes, together with the links between these. Our logic model was developed iteratively through several group interviews with programme staff, with the developing logic model progressively serving as the focus for discussion.

### Initial planning of quantitative analyses

Having developed the model, we then considered how to test whether and how the team was having the effects anticipated. The new team's capacity was small relative to the likely number of homeless patients, making it feasible to base an evaluation partly on a comparison between patients who received the Pathway intervention and patients who would have been eligible but did not. As regards outcome measures, we were able to analyse the estimated effects of the programme on length of stay and on readmission – both of which, looking ahead to the results of logic modelling, were potentially important outcomes. (Because of the possibility that patients might move outside the SLaM catchment area and this might not be captured, we analysed both readmission to psychiatric wards operated by SLaM and, separately, readmission to any psychiatric ward in England.)

### Data-set construction

Both these analyses were based mainly on data taken from the Clinical Record Interactive Search (CRIS) system, which is an anonymised version of SLaM's electronic patient record database, containing a mixture of structured and unstructured data,^6,7^ and which may be accessed using the SQL programming language. We extracted all those admissions (a) to adult acute psychiatric wards (those treating 18- to 65-year-olds), (b) that included a period on a ward serving Lambeth or Southwark and (c) that led to a discharge between 1 February 2015 (the day that the team started operating) and 31 March 2018. The complete data-set was created by supplementing each combination of patient identifier, admission date and discharge date with (a) a variable indicating whether the patient concerned had received the Pathway intervention during the admission; (b) the length of stay (after removing days on extended leave from the ward); (c) the date of the first readmission to a SLaM psychiatric bed within the study period; (d) the date of death (where applicable); (e) the date of the first recorded move to an address outside the four boroughs served (where applicable); and (f) basic clinical and demographic data, including data on service use in the preceding period. The only variable not taken from CRIS was the date of first admission to any English mental healthcare provider: these data were taken from Hospital Episode Statistics (HES) and were made available under a data sharing agreement between SLaM's Biomedical Research Centre and NHS Digital. All data were extracted on 7 June 2018. SLaM readmissions were included up to 25 May 2018; HES data were only available up to 31 March 2017, so the analysis of national readmissions (see below) is based on a subset.

### Selection of observations for the analysis

The entire data-set comprised a set of hospital admissions, during each of which an individual patient either received the Pathway intervention or did not. So that we could use these data to estimate the effects of the Pathway intervention, we used selection as the main way of minimising the effect of differences between those who were included in the analysis and had received the intervention (the treated observations – ‘treatment' being used here in its statistical sense of receiving an intervention whose effect is of interest) and those who were included in the analysis and did not receive the intervention (the control observations).

The Pathway team had explicit eligibility criteria: admission to one of the relevant wards, having no address to which discharge could be arranged and having no care coordinator. The team would accept patients irrespective of right to statutory entitlements, nationality or local connection. However, it is possible that other, implicit factors also influenced referral and acceptance. Therefore, rather than simply selecting treated and control observations using the explicit criteria, we instead used mixed-effects logistic regression to estimate the probability of treatment (treatment here again being used in its statistical sense of receiving an intervention of interest). We then selected observations whose probability indicated that they had had a realistic chance of either receiving or not receiving the Pathway intervention (for details see below). This increases the overall similarity of case and control observations; but more specifically in the context of a regression analysis it means that any observed combination of covariate values is likely to occur among both case and control observations (the so-called common support condition).^8^ Satisfying the common support condition helps to avoid estimation bias.

We worked with the entire data-set, as described above. Because homelessness was not reliably recorded, we developed a measurement strategy based on SQL processing of free-text progress notes. First, we counted the number of occurrences of the terms ‘NFA’, ‘no fixed abode’, ‘homeless’, ‘eviction’ and ‘being evicted’ across all notes made during the admission. Second, we used a receiver operating characteristics curve with programme participation as the dependent variable to define the count to use as a cut-off to determine programme eligibility. The resulting categorical variable was entered into the mixed-effects regression, along with allocation of a care coordinator, and an interaction term between the two, as well as age, gender, diagnosis, whether or not the person was detained under the Mental Health Act during the admission, longest admission in the previous year and number of discharges in the previous 2 years. (We did not include ethnicity and marital status in this regression analysis or the analyses of length of stay and readmission because of the presence of missing data; however, they are shown in Tables 1 and 2). The probability of programme participation was estimated based on the fixed effects in the model and the modal estimate of the subject-level random effect. We selected the subset with 0.1 < *P* < 0.9, eliminating observations for which common support was likely to be lacking (see above).^9^ The outcome analyses below were based on this subset.

**Table 1:** Characteristics of all hospital admissions involving the Pathway team and all other admissions^a^

Variable	All admissions involving the Pathway team (N=321)	All other admissions (N=5555)	*P*
Age, years: mean (s.d.)	37.1 (11.0)	39.4 (13.0)	0.002
Gender, *n* (%)			
Female	114 (36%)	2518 (45%)	0.001
Male	207 (64%)	3037 (55%)	
Marital status, *n* (%)			
Single	210 (79%)	4291 (81%)	0.036
Divorced/separated/widowed	35 (13%)	468 (9%)	
Married/cohabiting	21 (8%)	538 (10%)	
Ethnicity, *n* (%)			
White	139 (46%)	2132 (39%)	0.01
Any Black ethnicity	109 (36%)	2456 (45%)	
Any Other ethnicity	53 (18%)	864 (16%)	
ICD-10 diagnosis, *n* (%)			
F20 Schizophrenia	33 (11%)	1520 (28%)	<0.001
F21–F29 Other psychoses	76 (25%)	1382 (25%)	
F30–F31 Bipolar disorder and mania	23 (8%)	798 (15%)	
F32–F39 Depression	44 (14%)	429 (8%)	
F40–F48 Neurotic, anxious, stress-related	35 (11%)	222 (4%)	
F60–F69 Personality disorders	26 (9%)	596 (11%)	
F1x – Substance use disorders	363 (7%)	63 (21%)	
Other	5 (2%)	158 (3%)	
Legal status, *n* (%)			
Informal^b^	170 (53%)	1783 (32%)	<0.001
Section 2	88 (27%)	1846 (33%)	
Section 3 and forensic sections	63 (20%)	1926 (35%)	
Care coordinator during admission, *n* (%)	34 (11%)	3664 (66%)	<0.0001
Longest admission in the preceding year, days: mean (s.d.)	2.4 (14.7)	27.9 (93.2)	<0.0001
HoNOS item 11 at admission, *n* (%)			<0.001
0	54 (22%)	2237 (52%)	
1	28 (11%)	912 (21%)	
2	50 (20%)	641 (15%)	
3	43 (17%)	294 (7%)	
4	71 (29%)	227 (5%)	
ZZ99 address recorded during admission,^c^ *n* (%)			<0.001
Yes	201 (63%)	5121 (96%)	
No	118 (37%)	215 (4%)	
Count of homeless words or phrases in notes,^d^ *n* (%)			<0.001
None	1 (0%)	2762 (50%)	
1	2 (1%)	1084 (20%)	
2	5 (2%)	521 (9%)	
3	9 (3%)	333 (6%)	
4 or more	304 (95%)	855 (15%)	
Residential mobility during the admission or up to 1 month after discharge, *n* (%)			<0.001
No	134 (42%)	4574 (83%)	
Yes	184 (58%)	966 (17%)	

HoNOS item 11, Health of the Nation Outcome Scales, item ‘Problems with living conditions’.

a. The sample comprises admissions that included a period on a general adult ward and/or psychiatric intensive care unit serving Lambeth or Southwark, and which ended in discharge between February 2015 and March 2018. There were 5876 admissions overall, comprising 321 patients treated by the King's Health Partners Pathway Homeless Team and 5555 who were not. The *P*-values in the table derive from two-tailed *t*-tests for continuous variables and from overall χ²-tests for categorical variables.

b. Informal legal status includes patients initially detained under section 136 or section 4 of the Mental Health Act, but who were not made liable to detention under another section.

c. ZZ99 is a dummy postcode used in healthcare information systems in the UK to indicate various forms of non-standard residential status, primarily homeless. The Clinical Record Interactive Search (CRIS) database identifies as indicating homelessness the presence in an address of a postcode including ZZ99 or in which the first line is recorded as ‘homeless’ or ‘no fixed abode’.

d. The homeless text words and phrases counted in progress notes recorded during the admission were ‘homeless’, ‘NFA’, ‘no fixed abode’, ‘eviction’ or ‘being evicted’.

**Table 2:** Comparison of treated observations with control observations^a^

Variable	Treated observations (*n* = 280)	Control observations (*n* = 269)	*P*
Age, years: mean (s.d.)	37.7 (12.0)	37.2 (10.6)	0.55
Gender, *n* (%)			
Female	98 (35%)	105 (39%)	0.33
Male	182 (65%)	164 (61%)	
Marital status, *n* (%)			
Single	177 (78%)	188 (77%)	0.21
Divorced/separated/widowed	31 (14%)	26 (11%)	
Married/cohabiting	18 (8%)	30 (12%)	
Ethnicity, *n* (%)			
White	121 (46%)	98 (37%)	0.11
Black	94 (36%)	106 (40%)	
Other	48 (18%)	60 (23%)	
ICD-10 diagnosis, *n* (%)			
F20 Schizophrenia	31 (11%)	54 (20%)	0.001
F21–F29 Other psychoses	66 (24%)	73 (27%)	
F30–F31 Bipolar disorder and mania	21 (8%)	32 (12%)	
F32–F39 Depression	41 (15%)	23 (9%)	
F40–F48 Neurotic, anxious, stress-related	33 (12%)	18 (7%)	
F60–F69 Personality disorders	25 (9%)	20 (7%)	
F1x – Substance use disorders	58 (21%)	38 (14%)	
Other	5 (2%)	11 (4%)	
Legal status, *n* (%)			
Informal^b^	153 (55%)	84 (31%)	<0.001
Section 2	72 (26%)	86 (32%)	
Section 3 and forensic sections	55 (20%)	99 (37%)	
Longest admission in the preceding year, days: mean (s.d.)	1.5 (5.4)	3.8 (14.1)	0.01
HoNOS item 11 at admission, *n* (%)			0.003
0	46 (21%)	69 (31%)	
1	25 (12%)	40 (18%)	
2	46 (21%)	50 (22%)	
3	36 (17%)	33 (15%)	
4	62 (29%)	34 (15%)	
ZZ99 address recorded during admission,^c^ *n* (%)			<0.001
Yes	174 (62%)	217 (84%)	
No	105 (37%)	41 (16%)	
Residential mobility during the admission or up to 1 month after discharge, *n* (%)			0.01
No	109 (39%)	134 (50%)	
Yes	168 (61%)	134 (50%)	

a. The sample comprised admissions that included a period on a general adult ward and/or psychiatric intensive care unit serving Lambeth or Southwark, which ended in discharge between February 2015 and March 2018, and which were also in the subset used for analysis, with referral probability in the range 0.1 < *P* < 0.9. The *P*-values in the table derive from two-tailed *t*-tests for continuous variables and from overall χ²-tests for categorical variables.

b. Informal legal status includes patients initially detained under section 136 or section 4 of the Mental Health Act, but who were not made liable to detention under another section.

c. ZZ99 is a dummy postcode used in healthcare information systems in the UK to indicate various forms of non-standard residential status, primarily homeless. The Clinical Record Interactive Search (CRIS) database identifies as indicating homelessness the presence in an address of a postcode including ZZ99 or in which the first line is recorded as ‘homeless’ or ‘no fixed abode’.

### Length of stay

We constructed a linear regression of length of stay, using robust standard errors. Age, detained status, diagnosis and the length of the longest admission ending in the preceding year were included as covariates, because they had been found to have effects in a previous analysis in the same population;^10^ gender was included because it has been consistently found to be associated with length of stay in American studies.^11^ A functional form for continuous variables was defined using fractional polynomial transformations^12^ and an indicator variable was included for those who had had no admission in the preceding period.

### Readmission to SLaM wards

For the analysis of local readmissions, periods out of hospital were censored at the point that address data indicated a move outside the area covered by SLaM. Deaths were also treated as censoring events. Diagnosis, legal status, the length of the index admission and the number of discharges in the preceding 2 years were included as covariates because they had been found to have effects in a previous analysis in the same population.^13^ Age and gender were included based on results of a systematic review.^14^ Reparameterisation of diagnosis into psychotic/personality disorder/other non-psychotic was performed to reduce the number of parameters entering the analyses to no more than the number of readmissions divided by ten, therefore reducing the risk of overfitting.^15,16^ Multivariable fractional polynomial regression was used to select an appropriate functional form for age; length of stay seemed to be overinfluenced by a small number of observations, so was instead categorised. A full multivariable Cox regression model was used to estimate the effect of programme participation.

### Readmission to any English psychiatric ward

For the analysis of HES readmissions, we further restricted the data-set to admissions leading to discharge up to 31 March 2017, as readmission data were not available after that date. Periods out of hospital were censored only on death, if this occurred. Because of the more limited number of readmissions and greater risk of overfitting, gender was not included in the full model, as it had previously been found to be unassociated with readmission in the same population,^12^ and diagnosis was dichotomised as psychotic or non-psychotic. The analysis was otherwise identical to that of SLaM readmissions.

### Ethics and consent

We treated our study as a service evaluation rather than as research, in that its primary goal was not to generate generalisable theoretical knowledge. The logic model was created by programme staff, some of whom subsequently contributed to the writing of this paper (Z.K., S.K., R.R.) and others of whom are acknowledged below. All CRIS data for the quantitative study are fully anonymised and may be used without consent. (CRIS was approved on this basis as a data-set for secondary analysis by the Oxfordshire Research Ethics Committee C (08/H0606/71).)

## Results

### Logic model

**Fig. 1:**
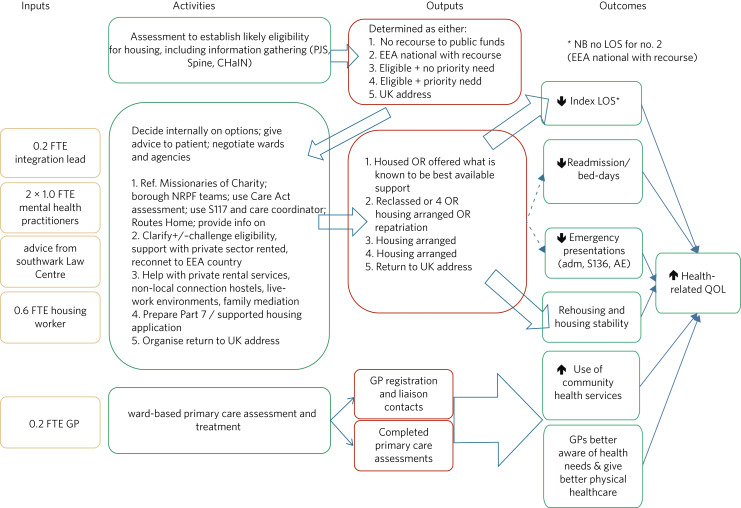
The final logic model. adm, admission; AE, accident and emergency department; CHaIN, Combined Homelessness and Information Link; EEA, European Economic Area; FTE, full-time equivalent; GP, general practitioner; LOS, length of stay; NRPF, no recourse to public funds; PJS, Patient Journey – SLaM's electronic patient record system; QOL, quality of life; S117, S136, sections 117 and 136 of the Mental Health Act 1983; Spine, the NHS Spine – a system providing a central record of NHS registration information.

Four versions of the logic model were produced: during the 1st, 8th, 15th and 20th month of the project's operation. Fig. 1 shows the last of these, produced on the 19 October 2016, reflecting four cycles of discussion.

The logic model makes use of the typical elements of inputs, activities, outputs and outcomes. It is a Z-design, with outputs of an initial stage (the results of an assessment for housing eligibility) entering into most of the subsequent activities of the team. Inputs are represented only by the staffing of the team (there were of course overhead costs and costs associated with the management of the team within the broader organisation). Activities may broadly be considered as the provision of expert support and liaison with other agencies such as general practitioners (GPs), community health services, social services, local authority housing departments, hostels, outreach teams and a wide range of community and voluntary sector services. However, the logic model contains several specific interventions. The model's outputs were housing, provision of alternative support, repatriation (for refugees, asylum seekers and other migrants) and reconnection, for example to primary care services.

Finally, the model – which was developed before any quantitative analysis – covers the hoped-for outcomes of the programme. These were considered more or less certain by respondents. Thus, solid arrows in Fig. 1 represent mechanisms that were thought to be more likely and dashed lines represent more tentative assertions. It was thought most likely that programme participation would reduce length of stay, would promote rehousing and housing stability and would promote the use of physical healthcare.

The quantitative analyses presented here therefore test only two small parts of this logic model, seeing whether the effects on length of stay and readmission are consistent with what was anticipated.

### Descriptive data on programme participants and non-participants

In total, there were 5876 admissions involving time on general adult wards serving Lambeth or Southwark that ended between February 2015 and March 2018. Of these, 321 were referred to the team and 5555 were not. Table 1 shows the characteristics of all those hospital admissions during which the Pathway team had been involved and all those admissions in which the Pathway team was not involved, using *t*-tests for differences in means and χ²-tests for differences in the distribution of categorical variables. There were missing data for some variables, notably marital status and the Health of the Nation Outcome Scales (HoNOS) housing variable. Referred patients tended to be younger, were more likely to be male, less likely to come from any kind of Black ethnicity, more likely to have a non-psychotic diagnosis, less likely to be detained under the Mental Health Act and had spent less time in hospital. They had more evidence of housing difficulties based on address data, HoNOS scores and prevalence of text terms related to homelessness, and were more likely to have recorded residential mobility during the admission or the month after discharge. As expected from the referral criteria for the team, they were much less likely to have a recorded care coordinator during the admission. Over the study period, the median number of patients taken on by the team was nine per month (interquartile range 7–10). A linear regression of monthly counts with robust standard errors indicated no change in activity over time (*P* = 0.12).

**Fig. 2:**
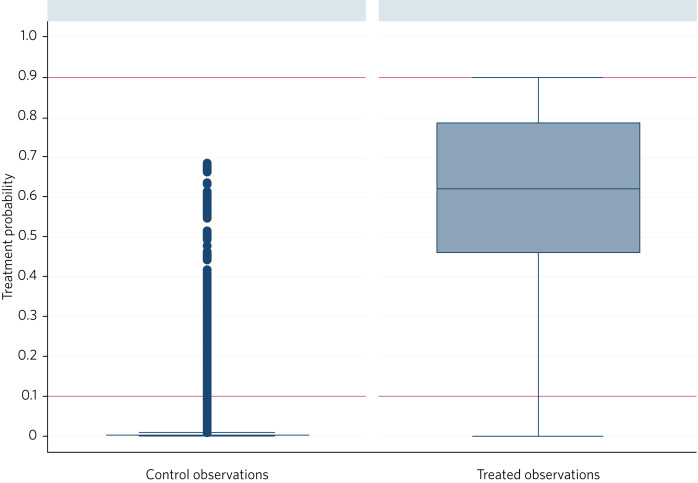
Estimated treatment probability comparing treated and control observations.

Figure 2 shows the distribution of the estimated probability of treatment, comparing all those admissions in which the Pathway intervention was delivered (*n* = 321) and all those in which it was not (*n* = 5555). Of these, there were 280 admissions during which the Pathway intervention was delivered whose probability of treatment was in the range 0.1 < *P* < 0.9, and 269 admissions during which the intervention was not given and where the treatment probability was in the same range. These comprised the treated and control observations for the analyses of length of stay and readmission.  It should be noted that many control observations fall outside the range of interest: inclusion of these would likely have introduced bias.

Table 2 is the analogue of Table 1, but compares only the treated and control observations, as defined above. Even after control selection, there were significant differences between treated patients and controls in the distribution of several variables: diagnosis, legal status, longest admission in the preceding year and three variables indicating homelessness or residential mobility. However, differences in age, gender and number of discharges in the preceding 2 years were not statistically significant.

### Analysis of length of stay

Analyses of length of stay were based on the total sample of 549 observations. The estimate of the effect of programme participation without adjustment for other factors was −32.0 days (95% CI −45.9 to −18.1; *P* < 0.001). The full model included the log of the length of the longest previous admission and a linear effect of age, alongside dummy variables for gender, diagnosis, legal status on admission and whether there had been any day in hospital in the preceding year. The adjusted effect of programme participation was estimated as −20.3 days (95% CI −32.5 to −8.1; *P* = 0.0012). Of the covariates in the analysis, only the effects of diagnosis reached a conventional level of statistical significance (*P* < 0.0001).

### Analysis of readmissions to SLaM wards

Again the analysis was based on the total 549 observations, which yielded 153 readmissions. The unadjusted hazard ratio for readmission was 0.69 (95% CI 0.49–0.97; *P* = 0.03). The adjusted hazard ratio was 0.82 (95% CI 0.58–1.17; *P* = 0.28), with only the effects of age (*P* = 0.0001) and number of previous admissions (*P* = 0.0002) being significant in this model.

### Analysis of admissions to any English psychiatric ward

Because of limited national readmission data, this analysis was based on 200 of the treated observations and 199 of the control observations, a total sample of 399 observations, which yielded 74 readmissions. The unadjusted hazard ratio for readmission was 0.65 (95% CI 0.40–1.07; *P* = 0.09). The adjusted hazard ratio was 0.70 (95% CI 0.42–1.16; *P* = 0.16). Only diagnosis entered significantly into the adjusted model (*P* = 0.04).

## Discussion

The evaluation findings presented here comprise a logic model for the KHP Pathway Homeless Team coupled with limited but promising evidence for programme outcomes that largely concur with that model. On the one hand, the assertion that the programme would reduce length of stay was entirely borne out by the quantitative data. On the other, the uncertainty that programme staff expressed regarding effects on readmission was shown to be justified. Although we do not include any element of economic evaluation at this stage, it would seem likely that the effects on length of stay alone would yield significant cost savings. The logic model expresses our best understanding of the mechanism of the effect on length of stay. Members of the team were able to delineate a series of more or less definite steps that they took in those scenarios that they typically encountered. Effects of the programme on length of stay would be expected to have arisen from the effect of such actions, whether this effect was the expedition of the provision of housing and therefore discharge or, alternatively, the expedition of discharge without rehousing, in those cases where the latter was an unattainable goal.

This broadly positive conclusion is in line with previous evidence supporting the value of specialist homeless healthcare teams in improving health and housing outcomes for patients in hospitals providing acute physical care.^17–19^ In service evaluations of the Pathway Homeless Teams established in acute hospitals, there appeared to be an immediate reduction in average bed-days for homeless patients, although this average may then have increased as the case-load of the teams came to be dominated by patients with more complex problems.^17^

### Strengths and limitations

The evaluation was limited by our ability to look at only two quantitative outcomes. Some other outcomes could, in principle, have been measured. We did not attempt to measure outputs and activities themselves. Qualitative interviews, perhaps focusing on specific cases and including homeless in-patients themselves, might have allowed a deeper interrogation of the theories that were put forward in the group interviews that led to the logic model. The evaluation does, however, have the merit of being based on a ‘theory-driven’ rather than ‘black-box’ approach:^20^ The effects we found can be interpreted within a logic model that was created based on expert practitioner knowledge, rather than standing in isolation.

The estimation and interpretation of programme effects based on non-experimental data needs to be cautious, but has a sound theoretical basis.^8^ The central issue is appropriate control selection, and here we were assisted by knowing the team's eligibility criteria and by the variety of data available to us. Estimation of the probability of referral to the team allowed us to show that we had not included observations whose treatment probability was either too high or too low. We attempted to address any remaining risk of bias using regression, following established methods. Here, more than in the case of control selection, we were limited by available data: in particular, there was little information on case complexity, which could conceivably have differed between treated and control observations. However, if anything, patients with more complex problems would have preferentially been selected for the Pathway intervention, which would be expected to reduce its apparent effect in our analysis. Information bias in the analysis of readmission to SLaM wards was addressed with an analysis of national readmissions.

### Implications and future research

The evidence presented here suggests quite strongly that the KHP Pathway Homeless Team had important positive effects on length of stay among programme participants. Other effects of the programme remain unexamined or partially examined. These findings certainly provide some justification for continued funding of the programme itself and could provide a justification for the piloting of similar approaches elsewhere, given the importance of the underlying health needs. However, it is also to be hoped that our findings will provide a stimulus to further attempts at evaluation. There is certainly a need to extend our findings to cover other outcomes. There is also a need to identify the more and less important components of the intervention, perhaps using a more sophisticated blend of qualitative and quantitative techniques. Finally, there is the issue of context. In many cases, interventions that work in one place do not work in the same way elsewhere, and further evaluation looking across multiple sites might help to uncover the context–mechanism–outcome configurations responsible for this,^21^ enabling modification of the intervention so that it may be made more reliably effective across different settings.

## Data Availability

Data can be accessed on application to the National Institute for Health and Care Research (NIHR) Biomedical Research Centre. Data will be stored for ten years following publication.

## About the authors

**Alex D. Tulloch**, PhD, MRCP, MRCPsych, is a consultant psychiatrist with South London and Maudsley NHS Foundation Trust (SLaM), London, UK. **Zana Khan**, MMedSci, MB, ChB, MRCGP, is General Practitioner (GP) Lead with the Pathway Homelessness Team at SLaM, London, UK. **Nigel Hewett**, FRCGP, is the Medical Director of the Pathway charity, London, UK. **Sophie Koehne**, BA, OT(PGDip), MRCOT, was a mental health practitioner on the team at the time of the study and is now Occupational Therapy (OT) Fellow at the Pathway charity, London, UK. **Ranga Rao**, MD, FRCPsych, is a consultant psychiatrist with SLaM, London, UK.

## References

[1] Aldridge RW, Story A, Hwang SW, Nordentoft M, Luchenski SA, Hartwell G, et al. Morbidity and mortality in homeless individuals, prisoners, sex workers, and individuals with substance use disorders in high-income countries: a systematic review and meta-analysis. Lancet 2018; 391: 241–50. 29137869 10.1016/S0140-6736(17)31869-X PMC5803132

[2] Luchenski S, Maguire N, Aldridge RW, Hayward A, Story A, Perri P, et al. What works in inclusion health: overview of effective interventions for marginalised and excluded populations. Lancet 2018; 391: 266–80. 10.1016/S0140-6736(17)31959-1 29137868

[3] Office of the Chief Analyst. Healthcare for Single Homeless People. Department of Health, 2010.

[4] Hewett N, Halligan A, Boyce T. A general practitioner and nurse led approach to improving hospital care for homeless people. BMJ 2012; 345: e5999. 23045316 10.1136/bmj.e5999

[5] McLaughlin JA, Jordan GB. Using logic models. In Handbook of Practical Program Evaluation (2nd edn): 55–80. Jossey-Bass, 2010.

[6] Perera G, Soremekun M, Breen G, Stewart R. The psychiatric case register: noble past, challenging present, but exciting future. Br J Psychiatry 2009; 195: 191–3. 19721105 10.1192/bjp.bp.109.068452

[7] Perera G, Broadbent M, Callard F, Chang CK, Downs J, Dutta R, et al. Cohort profile of the South London and Maudsley NHS foundation Trust Biomedical Research Centre (SLaM BRC) case register: current status and recent enhancement of an electronic mental health record-derived data resource. BMJ Open 2016; 6(3): e008721. 10.1136/bmjopen-2015-008721 PMC4785292 26932138

[8] Angrist JD, Pischke JS. Mostly Harmless Econometrics: An Empiricist's Companion. Princeton University Press, 2009.

[9] Crump RK, Hotz VJ, Imbens GW, Mitnik OA. Dealing with limited overlap in estimation of average treatment effects. Biometrika 2009; 96: 187–99.

[10] Tulloch AD, Khondoker MR, Fearon P, David AS. Associations of homelessness and residential mobility with length of stay after acute psychiatric admission. BMC Psychiatry 2012; 12: 121. 22905674 10.1186/1471-244X-12-121 PMC3505156

[11] Tulloch AD, Fearon P, David AS. Length of stay of general psychiatric inpatients in the United States: systematic review. Adm Policy Ment Health 2011; 38: 155–68. 20924662 10.1007/s10488-010-0310-3

[12] Royston P, Sauerbrei W, Becher H. Modelling continuous exposures with a ‘spike’ at zero: a new procedure based on fractional polynomials. Stat Med 2010; 29: 1219–27. 20191601 10.1002/sim.3864

[13] Tulloch AD, David AS, Thornicroft G. Exploring the predictors of early readmission to psychiatric hospital. Epidemiol Psychiatr Sci 2016; 25: 181–93. 25703270 10.1017/S2045796015000128 PMC6998497

[14] Donisi V, Tedeschi F, Wahlbeck K, Haaramo P, Amaddeo F. Pre-discharge factors predicting readmissions of psychiatric patients: a systematic review of the literature. BMC Psychiatry 2016; 16: 449. 27986079 10.1186/s12888-016-1114-0 PMC5162092

[15] Harrell FE, Lee KL, Matchar DB, Reichert TA. Regression models for prognostic prediction: advantages, problems, and suggested solutions. Cancer Treat Rep 1985; 69: 1071–7. 4042087

[16] Harrell FE. Regression Modeling Strategies: With Applications to Linear Models, Logistic Regression and Survival Analysis. Springer, 2001.

[17] Dorney-Smith S, Hewett N, Khan Z, Smith R. Integrating health care for homeless people: experiences of the KHP pathway homeless team. Br J Healthc Manag 2016; 22: 215–24.

[18] Blackburn RM, Hayward A, Cornes M, McKee M, Lewer D, Whiteford M, et al. Outcomes of specialist discharge coordination and intermediate care schemes for patients who are homeless: analysis protocol for a population-based historical cohort. BMJ Open 2017; 7(12): e019282. 10.1136/bmjopen-2017-019282 PMC5736042 29247113

[19] Hewett N, Buchman P, Musariri J, Sargeant C, Johnson P, Abeysekera K, et al. Randomised controlled trial of GP-led in-hospital management of homeless people (‘Pathway’). Clin Med 2016; 16(3): 223–9. 10.7861/clinmedicine.16-3-223 PMC5922699 27251910

[20] Stame N. Theory-based evaluation and types of complexity. Evaluation 2004; 10: 58–76.

[21] Pawson R, Tilley N. Realistic Evaluation. Sage, 1997.

